# Optical Sensors and Methods for Underwater 3D Reconstruction

**DOI:** 10.3390/s151229864

**Published:** 2015-12-15

**Authors:** Miquel Massot-Campos, Gabriel Oliver-Codina

**Affiliations:** Department of Mathematics and Computer Science, University of the Balearic Islands, Cra de Valldemossa km 7.5, Palma de Mallorca 07122, Spain; goliver@uib.es

**Keywords:** 3D reconstruction, stereo vision, structured light, laser stripe, LiDAR, structure from motion, underwater robotics

## Abstract

This paper presents a survey on optical sensors and methods for 3D reconstruction in underwater environments. The techniques to obtain range data have been listed and explained, together with the different sensor hardware that makes them possible. The literature has been reviewed, and a classification has been proposed for the existing solutions. New developments, commercial solutions and previous reviews in this topic have also been gathered and considered.

## 1. Introduction

The exploration of the ocean is far from being complete, and detailed maps of most of the undersea regions are not available, although necessary. These maps are built collecting data from different sensors, coming from one or more vehicles. These gathered three-dimensional data enable further research and applications in many different areas with scientific, cultural or industrial interest, such as marine biology, geology, archeology or off-shore industry, to name but a few.

In recent years, 3D imaging sensors have increased in popularity in fields such as human-machine interaction, mapping and movies. These sensors provide raw 3D data that have to be post-processed to obtain metric 3D information. This workflow is known as 3D reconstruction, and nowadays, it is seen as a tool that can be used for a variety of applications, ranging from medical diagnosis to photogrammetry, heritage reports or machinery design and production [1,2]. Thanks to recent advances in science and technology, large marine areas, including deep sea regions, are becoming accessible to manned and unmanned vehicles; thus, new data are available for underwater 3D reconstruction.

Due to readily-available off-the-shelf underwater camera systems, but also to custom-made systems in deep-sea robotics, an increasing number of images and video are captured underwater. Using the recordings of an underwater excavation site, scientists are now able to obtain accurate 2D or 3D representations and interact with them using standard software. This software allows the scientist to add measurements, annotations or drawings to the model, creating graphic documents. These graphic documents help to understand the site by providing a comprehensive and thematic overview and interface with data entered by experts (pilots, biologists, archaeologists, *etc*.), allowing reasonable access to a set of heterogeneous data [3].

Most 3D sensors developed are designed to operate in air conditions, but the focus of this paper is in the 3D reconstruction of underwater scenes and objects for archeology, seafloor mapping and structural inspection. This data gathering can be performed from a deployed sensor (e.g., from an underwater tripod or a fixed asset), operated by a diver or carried by a towed body, a remotely-operated vehicle (ROV) or an autonomous underwater vehicle (AUV).

Other authors have already reviewed some topics previously mentioned, for example Jaffe *et al.* [4] surveyed in 2001 the different prospects in underwater imaging, foreseeing the introduction of blue-green lasers and multidimensional photomultiplier tube (PMT) arrays. An application of these prospects is shown in Foley and Mildell [5], who covered in 2002 the technologies for precise archaeological surveys in deep water, such as image mosaicking and acoustic three-dimensional bathymetry.

In [6], Kocak *et al.* outlined the advances in the field of underwater imaging from 2005 to 2008, basing their work on a previous survey [7]. Caimi *et al.* [8] wrote their survey in 2008 on underwater imaging, as well, and summarized different extended range imaging techniques, as well as spatial coherency and multi-dimensional image acquisition. Years later, Bonin *et al.* [9] surveyed in 2011 different techniques and methods to build underwater imaging and lighting systems. Finally, in 2013, Bianco *et al.* [10] compared structured light and passive stereo, focusing on close-range 3D reconstruction of objects for the documentation of submerged heritage sites.

Structure from motion and stereoscopy are also studied by Jordt [11], who reported in her PhD thesis (2014) different surveys on 3D reconstruction, image correction calibration and mosaicking.

In this survey, we present a review of optical sensors and associated methods in underwater 3D reconstruction. LiDAR, stereo vision (SV), structure from motion (SfM), structured light (SL), laser stripe (LS) and laser line scanning (LLS) are described in detail, and features, such as range, resolution, accuracy and ease of assembly, are given for all of them, when available. Despite sonar sensors being acoustic, a concise summary is also given due to their extended use in underwater, and figures are presented to be compared to optical systems.

This article is structured as follows: Section 2 presents the underwater environment and its related issues. Section 3 reviews the measuring methods to gather 3D data. Section 4 evaluates the literature and the different types of sensors and technologies. Section 5 shows some commercial solutions, and finally, in Section 6, conclusions are drawn.

## 2. The Underwater Environment

Underwater imaging [12] has particular characteristics that distinguishes it from conventional systems, which can be summarized as follows:

(1) Limited on-site accessibility, which makes the deployment and operation of the system difficult [13].
(2) Poor data acquisition control, frequently implemented by divers or vehicle operators untrained for this specific task [14].
(3) Insufficient illumination and wavelength-dependent light absorption, producing dim and monotone images [15]. Light absorption also causes darkening on image borders, an effect somewhat similar to vignetting.
(4) Water-glass-air interfaces between the sensor and the scene, modifying the intrinsic parameters of the camera and limiting the performance of the image processing algorithms [16,17,18], unless specific calibration is carried out [19,20].
(5) Significant scattering and light diffusion that limits the operational distance of the systems.

These distinguishing traits will affect the performance of underwater imaging systems. Particular attention is paid to the typical range, resolution and/or accuracy parameters for the systems discussed in the next sections.

Additionally, images taken in shallow waters (<10 m) can be seriously affected by flickering, which produces strong light fluctuations due to the sunlight refraction on a waving air-water interface. Flickering generates quick changes in the appearance of the scene, making basic image processing functions, like feature extraction and matching, which are frequently used by mapping software [21], more difficult. Although some solutions to this problem can be found in the literature [22], flickering is still a crucial issue in many submarine scenarios.

### 2.1. Underwater Camera Calibration

Camera calibration was first studied in photogrammetry [23], but it has also been widely studied in computer vision [24,25,26,27]. The use of a calibration pattern or set of markers is one of the most reliable ways to estimate a camera’s intrinsic parameters [28]. In photogrammetry, it is common to set up a camera in a large field looking at distant calibration patterns or targets whose exact location, size and shape are known.

**Figure 1:**
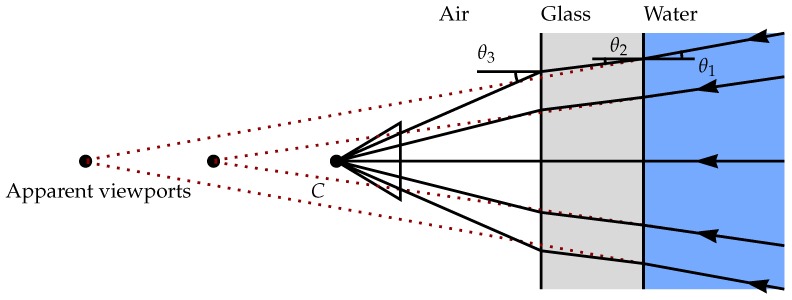
Refraction caused by the air-glass (acrylic)-water interface. The extension of the refracted rays (dashed lines) into air leads to several intersection points, depending on their incidence angles and representing multiple apparent viewpoints. Because of refraction, there is no collinearity between the object point in water, the center of projection of the camera and the image point [20].

Camera calibration is a major problem connected with underwater imaging. As mentioned earlier, refraction caused by the air-glass-water interface results in high distortion on images, and it must be taken into consideration during the camera calibration process [29]. This refraction occurs due to the difference in density between two media. As seen in Figure 1, the incident light beam passes through two media changes, modifying the light path.

According to Figure 1, the incident and emergent angles suffice for Snell’s Law, e.g.:

(1) $$r_{A}^{G}=\frac{\sin\theta_{3}}{\sin\theta_{2}}=\frac{n_{G}}{n_{A}}=1.49,\Rightarrow \theta_{3}>\theta_{2}$$

(2) $$r_{G}^{W}=\frac{\sin\theta_{2}}{\sin\theta_{1}}=\frac{n_{W}}{n_{G}}=0.89,\Rightarrow \theta_{2}<\theta_{1}$$

where $r_{A}^{G}$ is the refractive index between air and glass interfaces and $r_{G}^{W}$ is the refractive index between glass and water interfaces (for water $n_{W}=1.33$ at 20 ${}^{\circ }$C [30], for acrylic glass $n_{G}=1.49$ [31]).

If we replace Equation (2) in Equation (1),

(3) $$\frac{\sin\theta_{3}}{\sin\theta_{1}}=\frac{n_{W}}{n_{A}}=1.33\Rightarrow \theta_{3}>\theta_{1}$$

Therefore, the emergent angle $\theta_{3}$ is bigger than the incident angle $\theta_{1}$, causing the imaged scene to look wider than it is [14]. For planar interfaces, the deformation increases according to the distance from the center pixel of the camera, called pin-cushion distortion.

Changes in pressure, temperature and salinity alter the refraction index of water and even the camera handling, modifying the calibration parameters [32]. As a result, there is a mismatch between object-plane and image-plane coordinates. This problem has been addressed in two different ways: (1) developing new calibration algorithms that have refraction correction capability [29]; and (2) modifying existing algorithms to reduce the error due to refraction [33]. Other approaches, such as the one reported by Kang *et al.* [34], solve the structure and motion problem taking refraction into consideration.

According to Kwon [29], the refraction error caused by two different media can be reduced by considering radial distortion. Consequently, standard photogrammetric calibration software to calibrate the digital cameras and their housing can be used.

## 3. Measuring Methods

**Figure 2:**
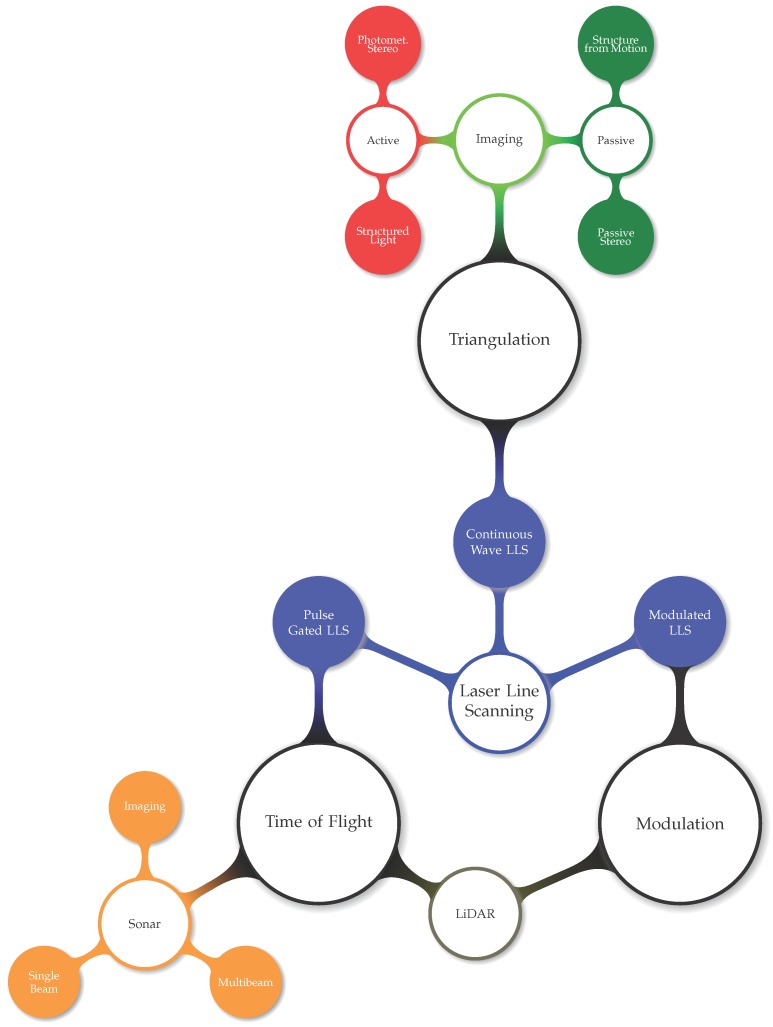
3D reconstruction sensor classification.

Sensors for three-dimensional measurement can be classified into three major classes depending on the measuring method: triangulation, time of flight and modulation. A sensor can belong to more than one class, which means that it uses different methods or a combination of them to obtain three-dimensional data, as depicted in Figure 2.

There is also another traditional classification method for sensing devices, active or passive, depending on how they interact with the medium. All of the methods in Figure 2 are active, except for passive imaging.

Active sensors are those that either illuminate, project or cast a signal with respect to the environment to help, enhance or measure the data to gather. An example of an active system is structured light, where a pattern is projected onto the object to reconstruct.

However, according to Bianco [10], those systems using artificial light sources, that are used just to illuminate the scene, but not for the triangulation of the 3D points, are considered passive.

Passive methods sense the environment with no alteration or change of the scene. An example of that is structure from motion, where image features are matched between different camera shots for a post-processed 3D triangulation. Camera-based sensors are the only ones that can be passive for 3D reconstruction, as the others are based on sound or on light projection.

### 3.1. Time of Flight

Time discrimination methods are based on controlling the travel time of the signal. By knowing the speed of the signal in the medium where it travels, the distance can be drawn. These methods achieve somewhat long distances, especially sonar, but in that case, extra care should be taken to prevent the measures from being affected by alterations in the sound speed, caused by water temperature, salinity and pressure changes.

At short distances, a small inaccuracy in the time measure can cause a great relative error in the result. Furthermore, some sensors require a minimum distance at which they can measure depending on their geometry.

Sonar, LiDAR and pulse gated laser line scanning (PG-LLS) are some examples of sensors using this principle to acquire 3D data.

### 3.2. Triangulation

Triangulation methods are based on measuring the distance from two or more devices (either signal sources or receivers) to a common feature or target with some known parameters.

For example, two cameras can obtain depth (e.g., a stereo rig) by searching in the image gathered by one camera features found in the other one. Once these features have been matched and filtered, the remaining features can be projected on the world as light rays coming from these two cameras. The triangle formed between the feature in the space and the two cameras is the basis for triangulation.

The limitation of triangulation sensors is the need for an overlapping region of the emitter field of view and the receiver one (or the two cameras in the stereo rig case) [17]. Besides, nearby features have a larger parallax, *i.e*., image disparity, than more distant ones, and as a consequence, the triangulation-based devices have a better *z* resolution for closer distances than for farther ones. Likewise, the bigger the separation of the cameras (baseline), the better is their *z* resolution.

Different techniques exist that compute 3D information by triangulation: structured light, laser stripe and photometric stereo (PhS) from active imaging, structure from motion and stereo vision from passive imaging and continuous wave laser line scanning (CW-LLS) from laser line scanning.

### 3.3. Modulation

While the time domain approach uses amplitude and time to discriminate multiple scattered, diffused photons, the frequency domain uses the differences in the amplitude and phase of a modulated signal to perform this task. The diffused photons that undergo many scattering events produce temporal spreading of the transmitted pulse. Only low frequency components are efficiently transmitted, whilst high frequency components are lost. This method has been reported in the literature both from airborne platforms and from underwater vehicles. They usually modulate the amplitude in frequencies in the order of GHz, thus requiring very sensitive sensors and accurate time scales. The receivers are usually photomultiplier tubes (PMT) or, more recently, photon counters made of avalanche photodiodes (APD). These sensors are generally triggered during a time window, and the incoming light is integrated. After the demodulation step, 3D information can be obtained from the phase difference.

It is known that coherent modulation/demodulation techniques at optical frequencies in underwater environments fall apart due to the high dispersion in the sea water path [6], as well as for the different absorption and scattering coefficients depending on the optical wavelength. Because there is a minimum for these coefficients in the blue-green region of the color spectra, amplitude modulation of the laser carrier of these wavelengths is the most used modulation technique in underwater reconstruction.

## 4. Sensors and Technologies

This section presents all of the sensors studied in this paper. At the end of each subsection, a table is presented indicating the accuracy and resolution values of the references listed, when available. Furthermore, if a value has been obtained from graphic plots, an approximate (≈) symbol has been used.

### 4.1. Sonar

The term sonar is an acronym for sound, navigation and ranging. There are two major kinds of sonars, active and passive.

Passive sonar systems usually have large sonic signature databases. A computer system uses these databases to identify classes of ships, actions (*i.e*., the speed of a ship or the type of weapon released) and even particular ships [35,36,37]. These sensors are evidently not used for 3D reconstructions; thus, they are discarded in this study.

Active sonars create a pulse of sound, often called a ping, and then listen for reflections of the pulse. The pulse may be at constant frequency or a chirp of changing frequency. If a chirp, the receiver correlates the frequency of the reflections to the known signal. In general, long-distance active sonars use lower frequencies (hundreds of kHz), whilst short-distance high-resolution sonars use high frequencies (a few MHz).

In the active sonar category, we can find three major types of sonars: multibeam sonar (MBS), single beam sonar (SBS) and side scan sonar (SSS). If the across track angle is wide, they are usually called imaging sonars (IS). Otherwise, they are commonly named profiling sonars, as they are mainly used to gather bathymetric data. Moreover, these sonars can be mechanically operated to perform a scan, towed or mounted on a vessel or underwater vehicle.

Sound propagates in water faster than in air, although its speed is also related to water temperature and salinity. One of the main advantages of sonar soundings is their long range, making them a feasible sensor to gather bathymetry data from a surface vessel, even for thousands of meters’ depth. At this distance, a resolution of tenths of meters per sounding is a good result, whilst if an AUV is sent to dive at an altitude of 40 m to perform a survey, a resolution of a couple of meters or less can be achieved.

One of the clearest examples of bathymetric data gathering is performed using MBS, as in [38]. This sensor can also be correlated to a color camera to obtain not only 3D, but also color information, as in [39], where its authors scan a pool using this method. However, in this case, its range is lowered to the visual available range.

MBS can also be mounted on pan and tilt systems to perform a complete 3D scan. They are usually deployed using a tripod or mounted on top of an ROV, requiring the ROV to remain static while the scan is done, like in [40].

A scanning SBS can carry out a 3D swath by rotating its head [41], as if it were a one-dimensional range sensor mounted on a pan and tilt head. The data retrieval is not as fast as with an MBS.

Profiling can also be done with SSS, which is normally towed or mounted in an AUV to perform a gridded survey. The SSS is mainly used on-board a constant speed vehicle describing straight transects. Even though SSS can be considered as a 2D imaging sonar, 3D information can be inferred from it, as depicted by Coiras *et al.* in [42].

Imaging sonars (IS) differ from MBS or SBS by a broadened beam angle (e.g., they capture a sonic image of the sea bottom instead of a thin profile). For instance, in [43], Brahim *et al.* use an imaging sonar with a field of view of 29 (azimuth) × 10.8 (elevation) to produce either 48 × 512 or 96 × 512 azimuth by-range images where each pixel contains the backscattered energy for all of the points in the scene located at the same distance with the same azimuth from the camera.

Other exotic systems have been researched, combining IS with conventional cameras to enhance the 3D output and to better correlate the sonar correspondences. In [44], Negahdaripour uses a stereo system formed by a camera and an imaging sonar. Correspondences between the two images are described in terms of conic sections. In [45], a forward looking sonar and a camera are used, and feature correspondences between the IS and the camera image are provided manually to perform reconstructions. Furthermore, in [46], an SfM approach from a set of images taken from an imaging sonar is used to recover 3D data.

The object shadows in a sonic image can also be used to recover 3D data, as in [47], where Aykin *et al.* are capable of reconstructing simple geometric forms on simple backgrounds. Its main requirement is that the shadow is distinguishable and that it lays on a known flat surface.

Beamforming (BF) is a technique aimed at estimating signals coming from a fixed steering direction, while attenuating those coming from other directions. When a scene is insonified by a coherent pulse, the signals representing the echoes backscattered from possible objects contain attenuated and degraded replicas of the transmitted pulse. It is a spatial filter that combines linearly temporal signals spatially sampled by a discrete antenna. This technique is used to build a range image from the backscattered echoes, associated point by point with another type of information representing the reliability (or confidence) of such an image. Modeling acoustic imaging systems with BF has also been reported by Murino in [48,49], where an IS of 128×128 pixels achieves a range resolution of ±3.5 cm. One pulse of this sonar system covers a footprint of 3.2×3.2 m${}^{2}$.

In [50], Castellani *et al.* register multiple MBS range measurements using global registration (ICP) with an average error of 15 cm.

Kunz *et al.* [51] fuse acoustic and visual information from a single camera, so that the imagery can be texture-mapped onto the MBS bathymetry (binned at 5 cm from 3 m), obtaining three-dimensional and color information.

Table 1 shows a comparison of the 3D reconstruction techniques using sonar.

**Table 1:** Summary of sonar 3D reconstruction solutions.

References	Sonar Type	Scope	Accuracy	Resolution
Pathak [38]	MBS	Rough map for path planning	≈1 m	2.5 cm
Rosenblum [52]	MBS	Small object reconstruction	-	≈8 cm
Hurtos [39]	MBS + Camera	Projects images on 3D surfaces	2.34 cm	-
Guo [41]	SBS	Small target 3D reconstruction	2.62 cm	-
Coiras [42]	SSS	Seabed elevation with UW pipe	19 cm	5.8 cm
Brahim [43]	IS	Sparse scene geometry	0.5 m	-
Aykin [47]	IS	Smooth surfaces 3D reconstruction	≈15 cm	1 cm
Negahdaripour [44,45,46]	IS + Camera	Alternative to stereo systems	≈5 cm	-

### 4.2. Light Detection and Ranging

Airborne scanning light detection and ranging (LiDAR) is widely used as a mapping tool for coastal and near shore ocean surveys. Similar to LLS, but surveyed from an aircraft, a laser line is scanned throughout the landscape and the ocean. Depending on the laser wavelength, LiDAR is capable of recovering both the ocean surface and the sea bottom. In this particular case, a green 532-nm laser that penetrates the ocean water over 30 m [53] is used in combination with a red or infrared laser. Both lasers return the echo from the sea surface, but only one reaches the underwater domain.

LiDAR has been used for underwater target detection (UWTD), usually mines, as well as for coastal bathymetry [54,55]. It is normally surveyed at heights of hundreds of meters (Pellen *et al.* survey mostly uniformly at 300 m [53]) with a swath of 100 to 250 m with a typical resolution in the order of decimeters. In [53], a resolution of 0.7 m is achieved. Moreover, the LiDAR signal can be modulated, enhancing its range capabilities and rejecting underwater backscatter [56,57].

Although this paper focuses on underwater sensors, LiDAR has been briefly mentioned, as it is capable of reconstructing certain coastal regions from the air. In Table 2, two 3D reconstruction references using LiDAR are compared.

**Table 2:** Summary of LiDAR 3D reconstruction solutions.

References	Class	Wavelength	LiDAR Model	Combination	Accuracy	Resolution
Reineman [53]	ToF	905 nm	Riegl LMS-Q240i	Camera, GPS	0.42 m	0.5 m
Cadalli [54]	ToF	532 nm	U.S. Navy prototype	PMT + 64 × 64 CCD	-	≈10 m
Pellen [55]	UWTD^1^	532 nm	ND:YAG laser	PMT	-	-
Mullen [56,57]	UWTD^1^	532 nm	ND:YAG laser	PMT + Microwave	-	-

${}^{1}$ Underwater target detection. No 3D reconstruction.

### 4.3. Laser Line Scanning

To increase the resolution of the systems exposed above, laser combined with imaging devices can be used. Green lasers working at 532 nm are a common solution as a light source because of their good trade-off between price, availability and low absorption and scattering coefficients in seawater. At the reception side, photomultiplier tubes (PMT) or photon counters can be used, although many approaches also use photodiodes or cameras.

For a larger operational range, preventing the effects of light scattering in the water, some LLS systems send out narrow laser pulses that will be gathered by range gated receivers.

There are three main categories of LLS: continuous wave LLS (CW-LLS), pulse gated LLS (PG-LLS) and modulated LLS. In Table 3, the reader can find a summary of the different LLS 3D reconstruction solutions. In addition to reconstruction, LLS are also used for long-range imaging (from ≈7 m). Some additional references are listed in Table 3, as well.

**Table 3:** Summary of laser line scanning 3D reconstruction solutions.

References	Aim	Type	Wavelength	Receiver	Accuracy	Resolution
Moore [58]	3D	CW-LLS	532 nm	Linescan CCD	-	1 mm
Moore [59]	3D	CW-LLS	532 nm	Linescan CCD	-	3 mm
McLeod [60]	3D	PG-LLS	-	-	7 mm	1 mm
Cochenour [61]	3D	Mod-LLS	532 nm	PMT	-	-
Rumbaugh [62]	3D	Mod-LLS	532 nm	APD	4.5 cm	1 cm
Dominicis [63]	3D	Mod-LLS	405 nm	PMT	5 mm	1 mm
Dalgleish [64]	Img.^1^	CW-LLS	532 nm	PMT	-	-
Dalgleish [64]	Img.^1^	PG-LLS	532 nm	PMT	-	-
Gordon [65]	Img.^1^	PG-LLS	488-514.5 nm	PMT	-	-
Mullen [66]	Img.^1^	Mod-LLS	532 nm	PMT	-	-

${}^{1}$ The technique is aimed at extended range imaging.

#### 4.3.1. Continuous Wave LLS

This subcategory uses a triangulation method to recover the depth. A camera-based triangulation device using a laser scan concept can be built using a moving laser pointer made of a mirror galvanometer and a line-scan camera, as shown in [58,59].

**Figure 3:**
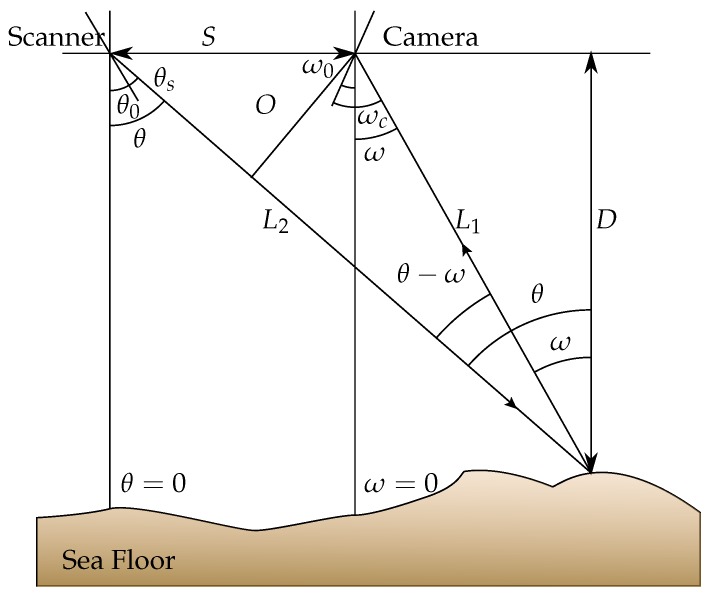
Triangulation geometry principle for a laser scanning system.

The geometric relationship between the camera, the laser scanner and the illuminated target spot is shown in Figure 3. The depth *D* of a target can be calculated from Equation (4).

(4) $$D=L_{1}\cos\left(\omega \right)$$

as:

(5) $$L_{1}=\frac{S\cos(\theta )}{\sin(\theta -\omega )}$$

since:

(6) $$\sin\left(\theta -\omega \right)=\frac{O}{L_{1}},\;\mathrm{and}\;O=S\cos\left(\theta \right)$$

therefore:

(7) $$D=\frac{S}{\tan(\theta )-\tan(\omega )}$$

where *S* is the separation (e.g., baseline) between the center of the scanning mirror and the center of the primary receiving lens of the camera (e.g., the center of perspective). Here, *θ* and *ω* are the scanning and camera pixel viewing angles, respectively.

The angles $\omega_{0}$ and $\theta_{0}$ are the offset mounting angles of the scanner and camera, and $\theta_{s}$ and $\omega_{c}$ are the laser beam angle known from a galvanometer or an encoder and the pixel viewing angle (with respect to the camera housing). Thus,

(8) $$\theta =\theta_{0}+\theta_{s}$$

(9) $$\omega =\omega_{0}+\omega_{c}$$

and:

(10) $$D=\frac{S}{\tan\left(\theta_{0}+\theta_{s}\right)-\tan\left(\omega_{0}+\omega_{c}\right)}$$

Both $\theta_{0}$ and $\omega_{0}$ have to be computed by calibration, so that afterwards, the distance to the target can be computed.

#### 4.3.2. Pulse Gated LLS

**Figure 4:**
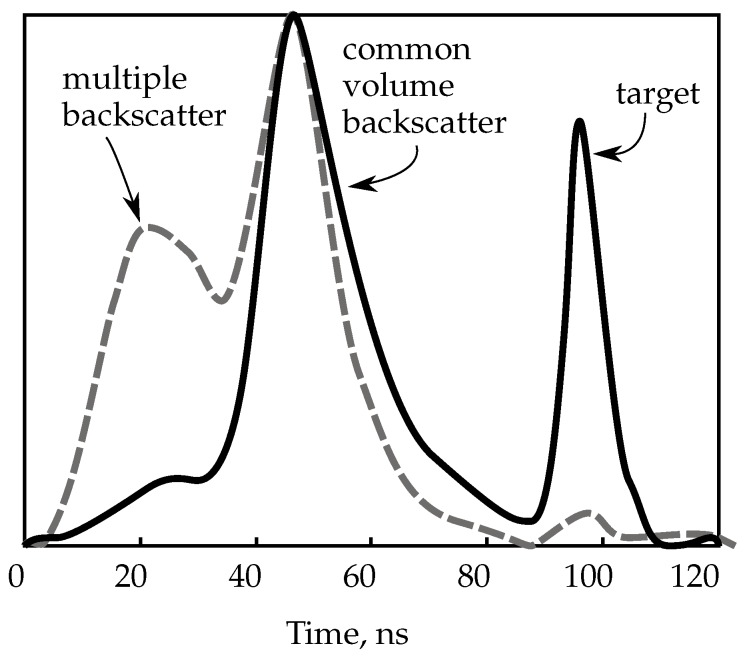
Representative normalized returning signal from an LLS. At higher turbidity (dashed gray line), the backscatter peak is stronger and the target return is weaker. The common volume backscatter is light that has been deflected once, whilst the multiple backscatter has been deflected twice or more times.

This ToF sensor has a simple principle: it illuminates a narrow area with a laser light pulse while keeping the receivers shutter closed. Then, it waits for the return of the light from the object by estimating its distance from the sensor and then opens the shutter so that only the light returning from the target is captured. For instance, in Figure 4, the shutter should have been opened from 80 to 120 ns to get rid of the unwanted backscatter.

This setup has been highly used in extended range imagery. In the early 1990s, the LLS system in [65] was used on the USS Dolphin research submarine and as a towed body to perform high resolution imagery at an extended range. This prototype used an argon ion gas laser, with a high power budget not available for most unmanned vehicles (ROVs or AUVs).

Dalgleish *et al.* [64] compared PG-LLS with CW-LLS as imaging systems. The experimental results demonstrate that the PG imager improved contrast and SNR (signal-to-noise ratio). Their sensor becomes limited by forward backscatter at seven attenuation lengths, whilst CW at six.

In true ToF 3D reconstruction, McLeod *et al.* [60] published a paper about a commercial sensor [67] mounted on the Marlin AUV. Their setup achieves an accuracy of 7 mm in a good visibility scenario, when measuring a point at 30 m.

#### 4.3.3. Modulated LLS

A modulated LLS characterizes the use of the frequency domain, instead of the spatial or time domain, to discern a change in the sent signal. In sonar chirps (radar as well), the modulation and posterior de-modulation of the signal give insight into the distance from the sensor to the target.

As stated before, amplitude modulation is the only realizable modulation in underwater scenarios. The original and the returned signal are subtracted, and the distance is obtained by demodulation of the remainder.

**Figure 5:**
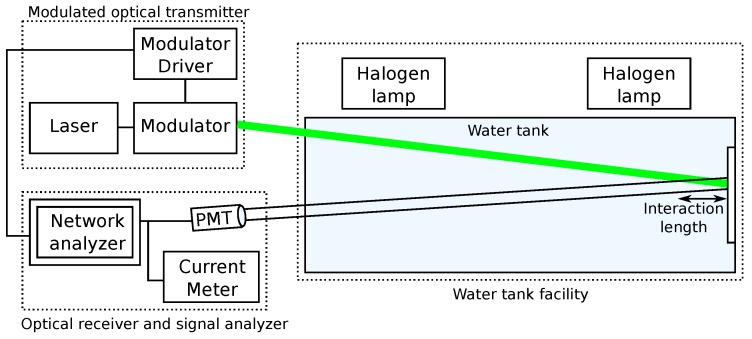
Laser line scanning setup including a modulated optical transmitter, an optical receiver and signal analyzer and a water tank facility. The interaction length is the distance over which the transmitted beam and the receiver field of view overlap. Reproduced from [66].

The same approach can be used for extended range imaging, as well, as seen in [66], where Mullen *et al.* have developed a modulated LLS that uses frequency modulation in the laser source in order to identify the distance at which the target has been illuminated. The optical modulation is used to discriminate scattered light. Different frequencies are compared experimentally, finding that a high frequency (90 MHz) reaches further than a lower one (50 MHz or 10 MHz). The setup used by the authors can be seen in Figure 5.

In [61], different modulation techniques based on ST-MP (single-tone modulated pulse) and PN-MP (pseudorandom coded modulated pulse) are compared for one-dimensional ranging. The results show that in clear water, the PN-MP stands as an improvement over the ST-MP due to the excellent correlation properties of pseudorandom codes.

In [62], a one-axis ranging solution is proposed. Although the authors characterize the solution as LiDAR, their setup is more similar to LLS, and the measurements are not taken from a plane. In the paper, a resolution of 1 cm from a distance of 60 cm is reported. This system could then be swept for a 3D reconstruction and work as a true LLS.

In [63], a simpler approach using an amplitude modulated blue laser (405 nm) at 80 MHz was used, called the MODEM-based 3D laser scanning system, that can reconstruct objects 8.5 meters away within a 5% of error. The system is similar to those described before, but this study focuses on the 3D reconstruction of the object, showing the potential of this technique for long-range underwater reconstruction.

### 4.4. Structured Light

These systems consist of a camera and a color (or white light) projector. The triangulation principle is used between these two elements and the projected object.

**Figure 6:**
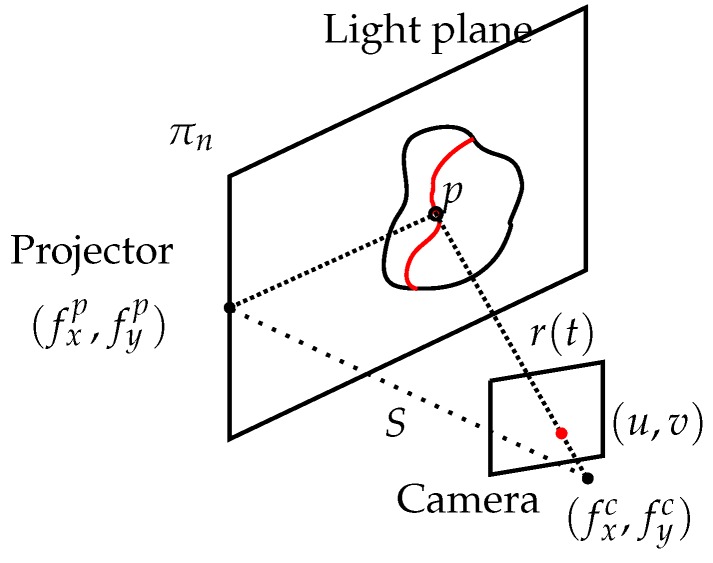
Triangulation geometry principle for a structured light system.

The projector casts a known pattern on the scene, normally a set of light planes, as shown in Figure 6, where both the planes and the camera rays are known. The intersection between them is unknown and can be calculated as follows.

Mathematically, a line can be represented in parametric form as:

(11) $$r\left(t\right)=\left\{\begin{array}{ccc} x & = & \frac{u-c_{x}}{f_{x}}\;t\\ y & = & \frac{v-c_{y}}{f_{y}}\;t\\ z & = & t \end{array}\right.$$

where $\left(f_{x},f_{y}\right)$ is the camera focal length in the *x* and *y* axes, $\left(c_{x},c_{y}\right)$ is the central pixel in the image and (u,v) is one of the detected pixels in the image. Supposing a calibrated camera and the origin in the camera frame, the light plane can be represented as in Equation (12).

(12) $$\pi_{n}:\;Ax+By+Cz+D=0$$

To find the intersection point, Equation (11) is substituted into Equation (12), giving Equation (13).

(13) $$t=\frac{-D}{A\frac{u-c_{x}}{f_{x}}+B\frac{v-c_{y}}{f_{y}}+C}$$

Different patterns have been used in the literature [68], even though it is a fact that binary patterns are the most used ones, because they are easy to achieve with a projector and simple to process. Binary patterns use only two states of light stripes in the scene, usually white light. At the beginning, there is only one division (black-to-white) in the pattern. In the following pattern projections, a subdivision of the previous pattern is projected until the software cannot segment two consecutive stripes. The correspondence of consecutive light planes is solved using time multiplexing. The number of light planes achievable with this method is fixed, normally to the resolution of the projector.

**Figure 7:**
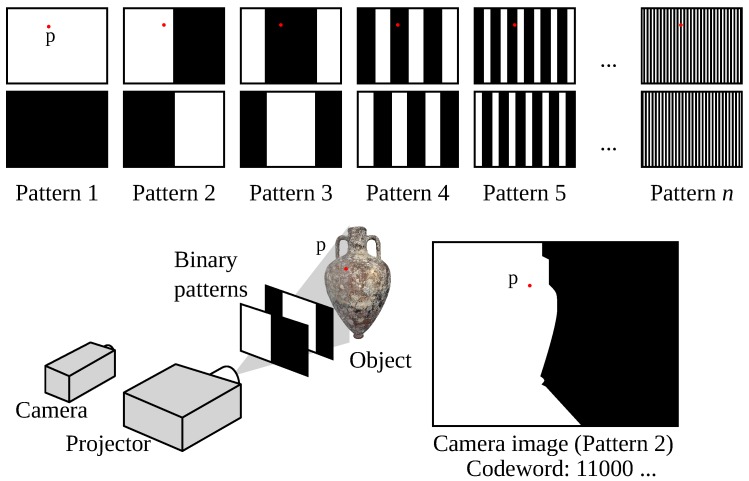
Binary structured light patterns. The codeword of a point *p* is created by the successive projection of patterns.

Time multiplexing methods are based on the codeword created by the successive projection of patterns onto the object surface (see Figure 7). Therefore, the codeword associated to a position in the image is not completely formed until all patterns have been projected. Usually, the first projected pattern corresponds to the most significant bit, following a coarse-to-fine paradigm. Accuracy directly depends on the number of projections, as every pattern introduces finer resolution in the image. In addition, the codeword basis tends to be small, providing resistance against noise [68].

On the other hand, phase shifting patterns use sinusoidal projections in the same operating mode to cover wider values in gray scale. By unraveling the phase value, different light planes can be obtained for just one state in the equivalent binary pattern. Phase shifting patterns are also time multiplexing patterns. Frequency multiplexing methods provide dense reconstruction for moving scenarios, but present high sensitivity to the non-linearities of the camera, reducing the accuracy and sensitivity to details on the surface of the target.

These methods use more than one projection pattern to obtain range information. De Bruijn sequences can achieve one-shot reconstructions by using pseudo-random sequences formed by alphabets of symbols in a circular string. If this theory is brought to matrices instead of vectors (e.g., strings), then those patterns are called *M*-arrays. These can be constructed by following a pseudo-random sequence [69]. Usually, these patterns use color to better distinguish the symbols in the alphabet. However, not all kinds of surface finishes and colors reflect correctly the incoming color spectra back to the camera [70,71]. One-shot coded patterns have also been used in air. However, to the best knowledge of the authors, there are no reports of these codification strategies in underwater scenarios.

In the literature, Zhang *et al.* project a grey scale four-step sinusoidal fringe [72]. Therefore, the pattern is a time multiplexing method using four different patterns. In their article, SL is compared to SV showing better behavior in SL on textureless objects. Similar results were obtained by Törnblom, projecting 20 different grey coded patterns in a pool [73]. An accuracy in the *z* direction of 2% was achieved with this system.

Bruno *et al.* [70] also project gray coded patterns with a final code shift of four pixel-wide bands. With these last shifts, better accuracy can be obtained compared to narrowing the pattern to only one pixel-wide patterns, where finding all of the thin black and white lines is more difficult. In this setup, a total of 48 patterns were used. However, this particular setup calculates the 3D points using the positions of two cameras determined during the calibration phase. The projector is used to illuminate the scene, whilst depth is obtained from the stereo rig. Thus, no lens calibration is needed for the projector, and any commercially-available projector can be used without compromising the accuracy of the measurements. This system would be a hybrid between SL and SV.

Another way to triangulate information using structured light is to sweep a light plane. This light plane can be swept either using the available pixels in the projector or by moving the projector. Narasimhan and Nayar [74] sweep a light plane into a tank with diluted milk and recover 3D information even in high turbidity scenarios where it is impossible to see anything but backscattering when using conventional floodlights. By narrowing the illuminated area to a light plane, the shapes of the objects in the distance can be picked out and therefore triangulated.

The use of infrared projectors, such as Kinect, has also been tested underwater [75]. The attempt confirmed that the absorption of the infrared spectrum is too strong to reach distances greater than a few centimeters.

#### Laser-Based Structured Light Systems

The systems presented in this section project laser light into the environment. Laser stripe (LS) systems are a subgroup of laser-based structured light systems (LbSLS), where although the pattern is fixed to be a line (a laser plane), the projector is swept across the field of view of the camera. Thus, for this setting, a motorized element is needed in addition to the laser if the system holding the camera and the laser is not moving. The relative position and orientation of the laser and camera system must be known in order to perform the triangulation process. The resolution of these systems is usually higher than stereoscopy, but they are still limited by absorption and scattering. The range of LS does not normally go over 3 m in clear waters [76], as will be seen later in the commercial solutions.

According to Bodenmann [77], the attenuation of light is significantly more pronounced in water than in air or in space, and so in order to obtain underwater images in color, it is typically necessary to be within 2 to 3 m of the seafloor or the object of interest. Moreover, these are some of the reported ranges for LS: 3 m for Inglis [76], 250 mm for Jakas [78] and 2 m for Roman [79].

Using an underwater stripe scanning system was initially proposed by Jaffe and Dunn in [80] to reduce backscattering. Tetlow and Spours [81] show in their article a laser stripe system with an automatic threshold setup for the camera, making this sensor robust to pixel saturation if the laser reflection is too strong. To that end, they programmed a table with the calibrated thickness of the laser stripe depending on the distance to the target, achieving resolutions of up to five millimeters at a distance of three meters.

Kondo *et al.* [82] tested an LS system in the Tri-Dog I AUV. Apart from using it for 3D reconstruction, they also track the image in real time to govern the robot. To keep a safe distance from the seabed, they center the laser line in the camera image by changing the depth of the vehicle. They report a resolution of 40 mm at three meters.

Hildebrandt *et al.* [83] mount a laser line onto a servomotor that can be rotated $45^{\circ }$ with an accuracy of $0.15^{\circ }$. The camera is a 640 × 480 CMOS shooting at 200 frames per second (fps) with a $90^{\circ }$ HFOV (horizontal field of view). The system returns 300k points in 2.4 seconds. Calibration is made in his article with a novel rig consisting of a standard checkerboard next to a grey surface on one side. The laser is better detected on a grey surface. On a white surface, light is strongly reflected, and the camera has to compensate for the vast amount of light by shortening the exposure time. The detection of the laser in the same plane of the calibration pattern is used to calculate the position of the laser sheet projector with respect to the camera.

In [84], a system consisting of a camera, a laser line and an LED light are mounted on the AUV Tuna Sand to gather 3D information, as well as imagery. The laser is pointed at the upper part of the image, whilst the lighting is illuminating the lower part. Therefore, there is enough contrast to detect the laser line. In [77,85,86], a similar system, called SeaXerocks (3D mapping device), is mounted on the ROV Hyper-Dolphin. With this system, the authors perform 3D reconstructions in real intervention scenarios, such as in hydrothermal sites and shipwrecks.

In [87], the Tuna Sand AUV is used with a different sensor. In this case, a camera and a motorized laser stripe are mounted in two independent watertight housings. By keeping the robot as static as possible, the laser is projected onto the scene whilst rotating it. Then, the camera captures the line deformation, from which the 3D information is recovered. In this paper, multiple laser scans from sea experiments at Kagoshima Bay are combined using the iterative closest point (ICP) algorithm. The reconstructed chimney is three meters tall at a 200-meter depth.

In [63,78], Jakas and Dominicis use a dual laser scanner to increase the field of view of a single laser stripe. The reported horizontal field of view is $180^{\circ }$. The system is very similar to the commercial sensor in [88]. They approximate the detected laser lines to be Gaussian and explain an optimization method to calibrate the camera-to-laser transformation. The authors claim that the achieved measuring error is below 4%.

Prats *et al.* [89,90,91] mount a camera fixed to the AUV Girona 500 frame and a laser stripe on an underwater manipulator carried by the vehicle. The stripe sweeps the scene by means of the robot arm, and the resulting point cloud is used to determine the target grasping points. The sea bottom is tracked to estimate the robot motion during the scanning process, so small misalignments between the data can be compensated.

Different approaches to the common laser stripe scanning have also been reported. In [92], two almost-parallel laser stripes are projected to compute the distance between these lines captured from a camera, to know the distance to the target. These values are used as an underwater rangefinder. However, 3D reconstruction was not the aim of the research.

In [93], Caccia mounts four laser pointers lined with a camera in an ROV. The four imaged pointers are used to calculate the altitude and the heading of the vehicle, assuming the seabed is flat.

Yang *et al.* mount a camera and a vertical laser stripe in a translation stage [94]. They recover 3D data interpolating from a data table previously acquired from calibration. Whenever a laser pixel is detected in the image, its depth value is calculated from the four closest points in the calibration data.

**Figure 8:**
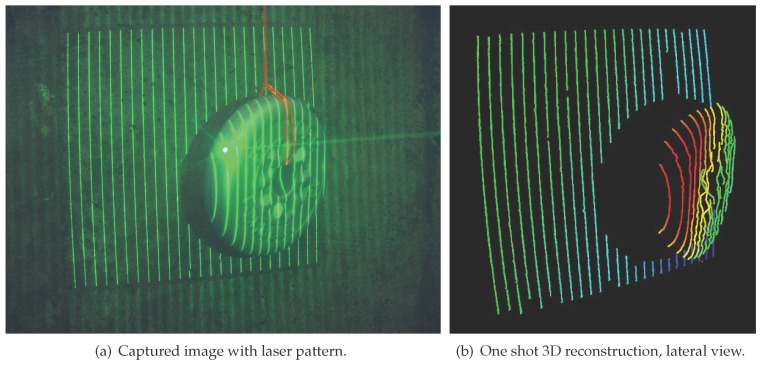
3D reconstruction of a 1-kg plate using LbSLS from [95].

Massot and Oliver [95,96,97], designed a laser-based structured light system that enhances simpler laser stripe approaches by using a diffractive optical element (DOE) to enhance a simple laser pointer, shaping the beam into 25 parallel lines, called a laser-based structured light (LbSL) system. The pattern is projected on the environment and recovered by a color camera. In one camera shot, this solution is capable of recovering sparse 3D information, as seen in Figure 8, whilst with two or more shots, denser information can be obtained. The system is targeted at underwater autonomous manipulation stages where a high density point cloud of a small area is needed, and during the manipulation, a one-shot and fast reconstruction aids the intervention.

In Table 4, the different SL references are compared. For the solutions with no clear results, the resolution has been deduced from the graphics in their respective articles.

**Table 4:** Summary of structured light 3D reconstruction solutions.

References	Type	Color/Wavelength	Pattern	Accuracy	Resolution
Zhang [72]	SL	Grayscale	Sinusoidal Fringe	≈1 mm	-
Tornblom [73]	SL	White	Binary pattern	4 mm	0.22 mm
Bruno [70]	SL	White	Binary pattern	0.4 mm	0.3 mm
Narasimhan [74]	SL	White	Light plane sweep	9.6 mm	-
Bodenmann [84,85]	LS	532 nm	Laser line	-	-
Yang [94]	LS	532 nm	Laser line	-	
Kondo [82]	LS	532 nm	Laser line	-	≈1 cm
Tetlow [81]	Mot. LS	532 nm	Laser line	1 cm	5 mm
Hildebrandt [83]	Mot. LS	532 nm	Laser line	-	-
Prats [89]	Mot. LS	532 nm	Laser line	≈1 cm	-
Nakatani [87]	Mot. LS	532 nm	Laser line	≈1 cm	-
Jakas [63,78]	Dual LS	405 nm	Laser line	See [88]	≈1 cm
Massot [96]	LbSL	532 nm	25 laser lines	3.5 mm	-

### 4.5. Photometric Stereo

**Figure 9:**
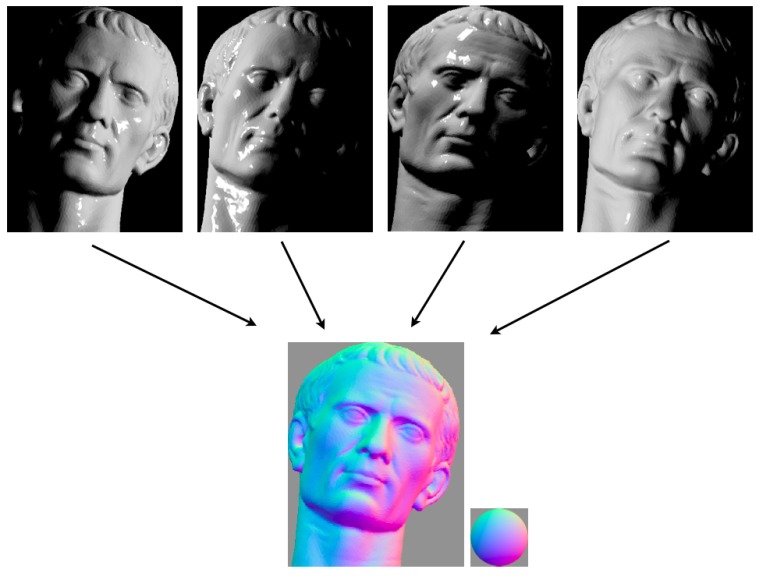
Photometric stereo setup: four lights are used to illuminate an underwater scene. The same scene with lighting from different sources results in the images used to recover three-dimensional information [99].

In situations where light stripe scanning takes too long to be practical, photometric stereo provides an attractive alternative. This technique for scene reconstruction requires a small number of images captured under different lighting conditions. In Figure 9, there is a representation of a typical PhS setup with four lights.

3D information can be obtained by changing the location of the light source whilst keeping the camera and the object in a fixed position. Narasimhan and Nayar present a novel method to recover albedo, normals and depth maps from scattering media [74]. Usually, this method requires a minimum of five images. In special conditions, such as the ones presented in [74], four different light conditions can be enough.

In [98], Tsiotsios *et al.* show that three lights are enough to compute tridimensional information. They also compensate the backscatter component by fitting a backscatter model for each pixel.

Like in time multiplexing SL techniques, PhS also suffers from long acquisition times; hence, these techniques are not suitable for moving objects. However, the cited references report them to be effective in clear waters for close range static objects.

### 4.6. Structure from Motion

SfM is a triangulation method that consists of taking images of an object or scene using a monocular camera. From these camera shots, image features are detected and matched between consecutive frames to know the relative camera motion and, thus, its 3D trajectory.

First, suppose a calibrated camera, where the principal point and calibration are known, as well as lens distortion and refractive elements to ensure an accurate 3D result.

Given *m* images of *n* fixed 3D points, then *m* projection matrices $P_{i}$ and *n* 3D points $X_{j}$ from the m·n correspondences $x_{ij}$ are to be estimated.

(14) $$x_{ij}=P_{i}X_{j},\;i=1,\cdots ,m,\;j=1,\cdots ,n$$

Therefore, if the entire scene is scaled by some factor *k* and, at the same time, the projection matrices by a factor of 1/k, the projection of the scene points remain the same. Thus, only with SfM, the scale is not available, although there are methods that compute it from known objects or by knowing the constraints of the robot carrying the camera.

(15) $$x=PX=\left(\frac{1}{k}P\right)\left(kX\right)$$

The one-parameter family of solutions parametrized by *λ* is:

(16) $$X\left(\lambda \right)=P^{+}x+\lambda c$$

where $P^{+}$ is the pseudo-inverse of P (*i.e*., $PP^{+}=I$) and c is its null-vector, namely the camera center, defined by Pc=0.

The approach of SfM is the least expensive in terms of hardware and the easiest to install in a real robot. Only a still camera or a video recorder is needed, with enough storage to keep a full dive in memory. Later, the images can be processed to obtain the required 3D models.

In the underwater medium, both feature detection and matching suffer from diffusion, non-uniform light and, eventually, sun flickering, making the detection of the same feature more difficult from different viewpoints. Depending on the distance from the camera to the 3D point, the absorption and scattering components vary, changing the colors and the sharpness of that particular feature in the image. More difficulties arise if images are taken from the air to the ocean [100].

Sedlazeck *et al.* show in [101] a real 3D scenario reconstructed from the ROV Kiel 6000 using an HD color camera. Features are selected using a corner detector based on image gradients. Later, the RANSAC [102] procedure is used to filter outliers after the features have been matched.

Pizarro *et al.* [103] use the SeaBED AUV to perform optical surveys, equipped with a 1280×1024 px CCD camera. The feature detector used is a modified Harris corner detector, and its descriptor is a generalized color moment.

In [104], Meline *et al.* compare Harris and SIFT features using a 1280×720 px camera in shallow water. In the article, the authors reconstruct a statue bust. They conclude that SIFT is not robust to speckle noise, contrary to Harris. Furthermore, Harris presented a better inlier count in the different scenarios.

McKinnon *et al.* [105] use GPU SURF features and a high resolution camera of 2272×1704 px to reconstruct a piece of coral. This setup presents several challenges in terms of occlusions of the different views. With their SfM approach, they achieve 0.7 mm accuracy at 1 to 1.5 m.

Jordt-Sedlazeck and Koch develop a novel refractive structure from motion algorithm that takes into account the refraction of glass ports in water [106]. By considering the refraction coefficient between the air-glass-water interface, their so-called refractive SfM improves the results of generic SfM.

Cocito *et al.* [107] use images captured by divers that always contain a scaling cube to recover scaled 3D data. The processing pipeline requires an operator to outline silhouettes of the area of interest of the images. In the case of the application in that paper, they were measuring bryozoan colonies’ volume.

In [108], the documentation of an archaeological site where experimental cleaning operations were conducted is shown. A commercial software, Photoscan by Agisoft, was used to perform a multi-view 3D reconstruction.

Nicosevici *et al.* [109] use SIFT features in a robotics approach, with an average error of 11 mm.

Ozog *et al.* [110] reconstruct a ship hull from an underwater camera that also acts as a periscope when the vehicle navigates on surface. Using SLAM and a particle filter, they achieve faster execution times (compared to FabMap). The error distribution achieved has a mean of 1.31 m and a standard deviation of 1.38 m. However, using planar constraints, they reduced the mean and standard deviation to 0.45 and 0.19 m, respectively.

The solutions presented are summarized in Table 5. Known reference distances must be visible in the images to recover the correct scale. In the solutions where a result is given, the authors have manually scaled the resulting point cloud to match a particular feature or human-made object.

**Table 5:** Summary of structure from motion 3D reconstruction solutions.

References	Feature	Matching Method	Accuracy	Resolution
Sedlazeck [101]	Corner	KTL Tracker	-	-
Pizarro [103]	Harris	Affine invariant region	3.6 cm	-
Meline [104]	Harris	SIFT	-	-
McKinnon [105]	SURF	SURF	0.7 mm	-
Jordt-Sedlazeck [106]	-	KLT Tracker	-	-
Cocito [107]	Silhouettes	Manually	≈1 cm	-
Bruno [108]	SIFT	SIFT	4.5 mm	-
Nicosevici [109]	SIFT	SIFT	11 mm	-
Ozog [110]	SIFT	SIFT	0.45 m	-

### 4.7. Stereo Vision

Stereoscopy follows the same working principle as SfM, but features are matched between left and right frames of a stereo camera to compute 3D correspondences. Once a stereo rig is calibrated, the relative position of one camera with respect the other is known, and therefore, the scale ambiguity is solved.

The earliest stereo matching algorithms were developed in the field of photogrammetry for automatically constructing topographic elevation maps from overlapping aerial images. In computer vision, the topic of stereo matching has been widely studied [111,112,113,114,115], and it is still one of the most active research areas.

Suppose two cameras $C_{L}$ and $C_{R}$ and two similar features $F_{L}$ and $F_{R}$ in each camera image. To compute the 3D coordinates of the feature *F*, whose projection in $C_{L}$ is $F_{L}$ and in $C_{R}$ is $F_{R}$, we trace a line $L_{L}$ that crosses $C_{L}$ focal point and $F_{L}$ and another line $L_{R}$ that crosses $C_{R}$ focal point and $F_{R}$. If both cameras’ calibration are perfect, $F=L_{L}\cap L_{R}$. However, as camera calibration is usually solved by least squares, the solution is not always perfect. Therefore, the approximate solution is taken as the closest point between $L_{L}$ and $L_{R}$ [116].

**Figure 10:**
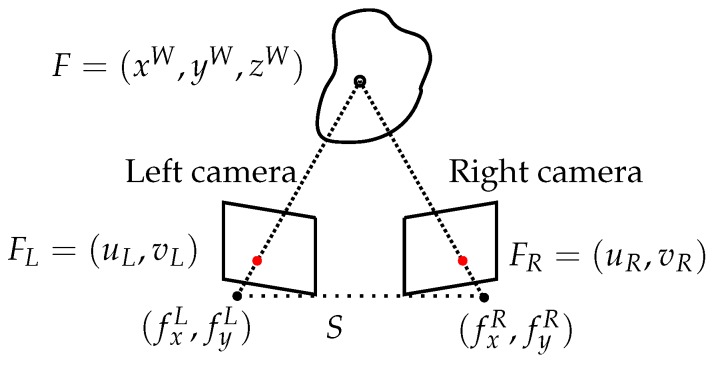
Triangulation geometry principle for a stereo system.

By knowing the relative position of the cameras and the location of the same feature in both images, the 3D coordinates of the feature in the world can be computed by triangulation. In Figure 10, the corresponding 3D point of the image coordinates $x=(u_{L},v_{L})$ and $x^{\prime }=\left(u_{R},v_{R}\right)$ is the point $p=(x^{W},y^{W},z^{W})$, which can also be written as $x^{\prime }Fx=0$ where *F* is the fundamental matrix [116].

Once the camera rig is calibrated (known baseline, relative pose of the cameras and no distortion in the images), 3D imaging can be obtained calculating the disparity for each pixel, e.g., perform a 1D search for each pixel in the left and right images, where block matching is normally used. The disparity is the difference in pixels from the left to the right image, where the same patch has been found; so, the depth *z* is given by:

(17) $$z=\frac{f\cdot b}{d}$$

where *d* is the disparity in pixels, *f* is the focal distance in pixels, *b* is the baseline in meters and *z* is the depth or distance of the pixel perpendicular to the image plane, in meters.

Once these 3D data have been gathered, the registration between consecutive frames can be done using 2D or 3D features or even 3D registration methods, such as ICP.

Fairly different feature descriptors and matchers have been used in the literature. SIFT [117,118,119,120,121,122] is one of the most used, as well as SURF [123], or even direct 3D registration with SIFT 3D [118] or ICP [117]. For instance in [124], Servos *et al.* perform refractive projection correction on depth images generated from a Bumblebee2 camera (12-cm baseline). The results obtained with this correction have better accuracy and more pixel correspondences, compared to standard methods. The registration is directly done in the generated point cloud using ICP.

Schmidt *et al.* [120] use commercial GoPro cameras to set a 35-mm baseline stereo rig and perform micro bathymetry using SIFT features. They achieve a resolution of 3 mm in their reconstructions.

In [122], the stereo system IRIS is hung from the tip of the arm of the Victor6000 ROV. The system uses SIFT combined with RANSAC to discard outliers. After that, a sparse bundle adjustment is performed to correct the navigation to survey natural underwater objects.

In [125], Hogue *et al.* combine a Bumblebee stereo and a inertial unit housed in a watertight case, called Aquasensor. This system is used to reconstruct and register dense stereo scenes. The reconstruction shows high drift if the IMU is not used; thus, an erroneous camera model is assumed to be the cause of this inaccuracy. The system is used by the authors to perform a reconstruction of a sunken barge.

Beall *et al.* [123] use a wide baseline stereo rig and extract SURF features from left and right image pairs. They track these features to recover the structure of the environment after a SAM (smoothing and mapping) step. Then, the 3D points are triangulated using Delaunay triangulation, and the image texture is mapped to the mesh. This setup is applied to reconstruct coral reefs in the Bahamas.

**Figure 11:**
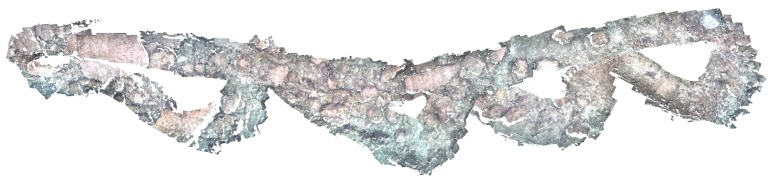
3D reconstruction from SV using graph SLAM (25×10 m, Mallorca) [127,131].

Negre *et al.* [126,127] perform 3D reconstruction of underwater environments using a graph SLAM approach in a micro AUV equipped with two stereo rigs. In Figure 11, a 3D reconstruction of Santa Ponça Bay is displayed, covering an area of 25×10 m.

Johnson-Roberson *et al.* [128] studied the generation and visualization of large-scale reconstructions using stereo cameras. In their manuscript, image blending techniques and mesh generation are discussed to improve visualization by reducing the complexity of the scene in proportion to the viewing distance or relative size in screen space.

Fused stereoscopy and MBS have been reported in [129]. There, Galceran *et al.* provide a simultaneous reconstruction of the frontal stereo camera and the downwards-looking MBS.

Another example of this set of sensors is shown by Gonzalez-Rivero [130], where its output is used to monitor a coral reef ecosystem and to classify the different types of corals.

Nurtantio *et al.* [119] use three cameras and extract SIFT features. The reconstruction of the multi-view system is triangulated using Delaunay triangulation. However, they manually preprocess the images to select whether they are suitable for an accurate reconstruction. The outlier removal stage is also manual.

Inglis and Roman constrain stereo correspondences using multibeam sonar [132]. From the Hercules ROV, navigation data, multibeam and stereo are preprocessed to reduce the error, and then, the sonar and optical data are mapped into a common coordinate system. They back project the range data coming from the sonar to the camera image and limit the available *z* correspondence range for the algorithm. To simplify this approach, they tile the sonar back projections into the image and generate tiled minimum and maximum disparity values for an image region (e.g., a tile). The number of inliers obtained with this setup increases significantly compared to an unconstrained system.

In Table 6, the different solutions are presented and compared.

**Table 6:** Summary of stereoscopy 3D reconstruction solutions.

References	Feature	Matching Method	Baseline	Accuracy	Resolution
Kumar [117]	SIFT	RANSAC and ICP	-	-	-
Jasiobedzki [118]	SIFT	SIFT3D and SLAM	-	-	-
Nurtantio [119]	SIFT	SIFT	8 and 16 cm	-	-
Schmidt [120]	SIFT	SIFT	35 mm	-	3 mm
Brandou [122]	SIFT	SIFT	-	-	-
Beall [123]	SURF	SURF and SAM	60 cm	-	-
Servos [124]	-	ICP	12 cm	26.4 cm	-
Hogue [125]	Corners	KLT tracker	12 cm	2 cm	-
Inglis [132]	SIFT	SIFT	42.5 cm	-	-

### 4.8. Underwater Photogrammetry

It is commonly accepted that photogrammetry is defined as the science or art of obtaining reliable measurements by means of photographs [133]. Therefore, any practical 3D reconstruction method that uses photographs (e.g., imaging-based methods) to obtain measurements are photogrammetric methods. Photogrammetry comprises methods of image measurement and interpretation often shared with other scientific areas in order to derive the shape and location of an object or target from a set of photographs. Hence, techniques such as structure from motion and stereo vision belong to both photogrammetric and computer vision communities.

In photogrammetry, it is common to set up a camera in a large field looking at distant calibration targets whose exact location has been precomputed using surveying equipment. There are different categories for photogrammetric applications depending on the camera position and object distance. For example, aerial photogrammetry is normally surveyed at a height of 300 m [134].

On the other hand, close-range photogrammetry applies to objects ranging from 0.5 to 200 m in size, with accuracies under 0.1 mm and around 1 cm at each end. In a close-range setup, the cameras observe a specific volume where the object or area to reconstruct is totally or partially in view and has been covered with calibration targets. The location of these targets can be known as before or calculated after the images have been captured if their shape and dimensions are known [134].

Image quality is a very important topic in photogrammetry. One of the main important fields of this community is camera calibration, a topic that has already been introduced in Section 2.1. If absolute metric accuracy is required, it is imperative to pre-calibrate the cameras using one of the techniques previously mentioned and to use ground control points to pin down the reconstruction. This is particularly true for classic photogrammetry applications, where the reporting of precision is almost always considered mandatory [135].

Underwater reconstructions can also be referred to as underwater photogrammetric reconstructions when they have a scale or dimension associated with the objects or pixels of the scene (e.g., if the resulting 3D model is metric) and if the data were gathered using cameras.

According to Abdo *et al.* [136], an underwater photogrammetric system for obtaining accurate measurements of complex biological objects needs to: (1) be suitable for working in restrictive spaces; (2) allow one to investigate relatively large areas carried out on one or numerous organisms; (3) admit the acquisition of data easily, performed *in situ* and efficiently; and (4) provide a measurement process that is easy to perform, precise, accurate and accomplished in a reasonable time lapse.

The most accurate way to recover structure and motion [137] is to perform robust non-linear minimization of the measurement (re-projection) errors, which is commonly known in the photogrammetry communities as bundle adjustment [28]. Bundle adjustment is now the standard method of choice for most structure-from-motion problems and is commonly applied to problems with hundreds of weakly calibrated images and tens of thousands of points. In computer vision, it was first applied to the general structure from motion problem and then later specialized for panoramic image stitching [28].

Image stitching originated in the photogrammetry community, where more manually-intensive methods based on surveyed ground control points or manually registered tie points have long been used to register aerial photos into large-scale photo-mosaics [23]. The literature on image stitching dates back to work in the photogrammetry community in the 1970s [138,139].

Underwater photogrammetry can also be associated with other types of measures, such as the measure of biological organisms’ volumes with 3D reconstruction using an stereo pair [136], the sustainability of fishing stocks [140], examining spatial biodiversity, counting fish in aquaculture [141], continuous monitoring of sediment beds [142] or to map and understand seabed habitats [13,21].

Zhukovsky *et al*. [143] reconstruct an antique ship, similar to [144]. In [32], Menna *et al.* reconstruct the sunken vessel Costa Concordia using photogrammetric targets to reconstruct and assess the damaged hull.

Photogrammetry is also performed by fusing data from diverse sensors, such as in [145], where chemical sensors, a monocular camera and an MBS are fused in an archaeological investigation, and in [146], where a multimodal topographic model of Panarea Island is obtained using a LiDAR, an MBS and a monocular camera.

Planning a photogrammetric network with the aim of obtaining a highly-accurate 3D object reconstruction is considered as a challenging design problem in vision metrology [147]. The design of a photogrammetric network is the process of determining an imaging geometry that allows accurate 3D reconstruction. There are very few examples of the use of a static deployment of cameras working as underwater photogrammetric networks [148] because this type of approach is not readily adapted to such a dynamic and non-uniform environment [149].

In [150], de Jesus *et al.* show an application of photogrammetry for swimming movement analysis with four cameras, two underwater and two aerial. They use a calibration prism composed of 236 markers.

Leurs *et al*. [151] estimate the size of white sharks using a camera and two laser pointers, with an accuracy of ±3 cm from a distance of 12 m.

Different configurations to monocular or stereo camera systems have also been reported. In [152], Brauer *et al.* use a stereo rig and a projector (SL). Using fringe projection, they achieve a measurement field of 200×250 mm and a resolution of 150 μm.

In [153], Ekkel *et al.* use a stereo laser profiler (four cameras, two for positioning with targets and two for laser triangulation) using a 640-nm laser. They report an accuracy of 0.05 mm in the object plane.

## 5. Commercial Solutions

There exist different commercial solutions for gathering 3D data or to help with calculating it. In Table 7, a selection of alternatives is shown.

Teledyne sells an underwater LLS called INSCAN [154]. This system must be deployed underwater or fixed to a structure. The device samples 1 m${}^{2}$ in 5 s at a 5-m range.

SL1 is a similar device from 3D at Depth [67]. In fact, this company worked with Teledyne in this design [155], and the specifications of these two pieces of equipment are quite close.

3DLS is a triangulation sensor formed by an underwater dual laser projector and a camera. It is produced by Smart light devices and uses a 15-W green laser.

2G Robotics has three models of triangulation-based laser scanners fitting different ranges [156,157,158]. These are motorized solutions, so they must be deployed and static during their scan.

Savante provides three products. Cerberus [159] is a triangulation sensor formed by a laser pointer and a receiver, capable of recovering 3D information. SLV-50 [160] is another triangulation sensor formed by a laser stripe and a high sensitivity camera, and finally, Lumeneye [161] is a laser stripe that only casts laser light on the scene.

Tritech provides (similar to Savante) a green laser sheet projector called SeaStripe [162]. The 3D reconstruction must be performed by the end-user camera and software.

**Table 7:** Available commercial solutions to perform 3D reconstruction.

Commercial Solutions		Range (m)		Depth	Resolution	Field of view	Motorized	Method
Name	Company	Min	Max	(m)	(mm)	(deg)		
INSCAN [154]	Teledyne CDL	2	25	3000	5	30×30×360	yes	TOF
SL1 [67]	3D at Depth	2	30	3000	4	30×30×360	yes	TOF
3DLS [88]	Smart Light Devices	0.3	2	4000	0.1	-	-	Triangulation
ULS-100 [156]	2g Robotics	0.1	1	350	1	50×360	yes	Triangulation
ULS-200 [157]	2g Robotics	0.25	2.5	350	1	50×360	yes	Triangulation
ULS-500 [158]	2g Robotics	1	10	3000	3	50×360	yes	Triangulation
Cerberus [159]	Savante	-	10	6000	-	-	-	Triangulation
SLV-50 [160]	Savante	-	2.5	6000	1	60	no	Triangulation
Lumeneye [161]	Savante	-	-	6500	-	65	no	Laser only
SeaStripe [162]	Tritech	-	-	4000	-	64	no	Laser only

## 6. Conclusions and Prospects

The selection of a 3D sensing system to be used in underwater applications is non-trivial. Basic aspects that should be considered are: (1) the payload volume, weight and power available, in case the system is an on-board platform, (2) the measurement time, (3) the budget and (4) the expected quality of the data gathered. Regarding the quality, optical sensors are very sensitive to water turbidity and surface texture. Consequently, factors, such as the target dimensions, surface, shape or accessibility, may influence the choice and adaptiveness of the sensor to the reconstruction problem. Table 8 presents a comparison of the solutions surveyed in this article according to its typical operative range, resolution, ease of use, relative price and its suitability to be used on different platforms.

Underwater 3D mapping has been historically carried out by means of acoustic multibeam sensors. In that case, the information is normally gathered as an elevation map, and more recently, color and texture can be added afterwards from photo-mosaics, if available.

Color or texture information must be acquired using cameras operating at relatively short distances (<5 m, typically) and with a low cruise speed. In general, mono-propeller AUVs are not appropriate for optical imaging applications, because they cannot slow down their speed as required by the optical equipments. On the other hand, hovering vehicles are suitable for imaging-based sensors, as they can adjust their velocity to the sensors’ needs. In some particular cases, even divers can be a choice.

Optical mapping can also be accomplished with only SfM and, as industrial ROVs most often incorporate a video camera, it is feasible to record the needed images and reconstruct an entire scene (see Campos *et al.* [163], for example). However, these reconstructions lack a correct scale, and they are computationally demanding. If, instead, a stereo rig is used, SV techniques can be applied and can solve the scale problem.

According to Bruno, SV is the easiest way to obtain the depth of a submarine scene [70]. These passive sensors are widely used because of their low cost and simplicity. Similarly to SfM, SV needs textured scenes to achieve satisfactory result, giving rise to missing parts corresponding to untextured regions in the final reconstruction.

**Table 8:** Strengths and weaknesses of the sensors and techniques for 3D reconstruction.

3D technique	Range	Platform	Resolution	Ease of assembly	Price
MBS	<11,000 m	V^1^, T^2^, ROV, AUV	Low	Intermediate	High
SBS	<6000 m	V, ROV, AUV	Low	Intermediate	High
SSS	<150 m	T, AUV	Low	Intermediate	High
IS	<150 m	V, T, ROV, AUV	Low	Intermediate	High
LiDAR	<20 m	Aerial	Low		High
CW-LLS	<10 m	ROV	Intermediate	Low	High
PG-LLS	<10 m	ROV	Intermediate	Low	High
Mod. LLS	<10 m	ROV	Intermediate	Low	High
SfM	<3 m	ROV, AUV	Intermediate	High	Low
SV	<3 m	ROV, AUV	Intermediate	Intermediate	Low
PhS	<3 m	ROV	Intermediate	Intermediate	Low
VW-SL	<3 m	ROV, AUV	High	Intermediate	Intermediate
CW-SL	<10 m	ROV, AUV	High	Intermediate	Intermediate

${}^{1}$ Vessel; ${}^{2}$ Towed.

To overcome the above-mentioned problems of SfM and SV and trying to increase the resulting resolution, SL uses light projection to cast features on the environment. These sensors are capable of working at short distances with high resolution, even for objects without texture. The drawback, compared to SV, is a slower acquisition time caused by the need to move the projection atop the scene or even to use different patterns. The acquisition time is a relevant problem that limits the use of SL systems in real conditions where the relative movement between the sensor and the scene can give rise to reconstruction errors.

In addition, acquiring data from dark objects using SL is, in general, strongly influenced by illumination and contrast conditions [70]. Shiny objects are also challenging for SL, because the reflected light may mislead the pattern decoder. Moreover, due to the large illuminated water volume, this technique is strongly affected by scattering, reducing its range.

To minimize absorption, as well as common volume scattering, LbSL systems take advantage of selected wavelength sources in the green-blue region of the spectrum, extending their capable range. For an improved reduction of the scattering effects, the receiver window can be narrowed as in LLS sensors; even more, the emitter and the receiver can also be pulse gated [64], even though this strategy can be limited by a contrast decline.

On the other hand, when a precise and closer look at an object or structure is needed, LLS technology is not always suitable, as it has a large minimum measuring distance.

Amongst optical solutions, laser-based sensors present a good trade-off between cost and accuracy, as well as an acceptable operational range. Accordingly, regarding the foreseeable future, more research on laser-based structured light and on laser line scanning underwater is needed. These new devices should be able to scan while the sensor is moving, just like MBS, so software development and enhanced drivers are also required.

Another challenge for the future is to develop imaging systems that can eliminate or reduce scattering while imaging. Solutions such as pulse gated cameras and laser emitters are effective [164], but still expensive.

Overall, it is quite clear that no single optical imaging system fits all of the 3D reconstruction needs, covering very different ranges and resolutions. Besides, it is important to point out the lack of systematic studies to compare, with as much precision as possible, the performance of different sensors on the same scenario and conditions. One of these studies is authored by Roman *et al.* [79], who compared laser-based SL to SV and MBS, mapping a small area of a real underwater scenario using an ROV. In that case, the stereo data showed less definition than the sonar and the SL. The comparison was made during a survey where laser images were collected at 3 Hz, at a speed of 2 to 5 cm/s from 3 m above the bottom, whilst stereo imagery was captured on a separate survey at 0.15 Hz at a speed of 15 cm/s and a distance of 1.5 to 3 m, giving a minimum overlap of 50%. MBS was captured during the laser survey at 5 Hz. As seen in these numbers, a different data rate induces less or more spatial resolution. Nonetheless, Roman *et al.* concluded that SL offers a high resolution mapping capability, better than SV and MBS for close-range reconstructions, such as the investigation of archaeological sites.

Massot *et al.* in [96] provide a systematic analysis comparing SV and LbSL in a controlled environment. To that end, a robot arm is used to move the sensors describing a precise path, surveying a 3×2 m underwater scene created in a water tank containing different objects of known dimensions. Apart from other numerical details, the authors conclude that for survey missions, stereo data may be enough to recover the overall shape of the underwater environment, whenever there is enough texture and visibility. In contrast, when the mission is aimed at manipulation and precise measurements of reduced areas are needed, LbSL is a better option.

It would be advisable to work on similar approaches to the aforementioned for the near future, contributing to a better knowledge of each individual sensor behavior when used in diverse situations and applications and also to the progress in multisensor data integration methodologies.

**Table 9:** Strengths and weaknesses of the sensors and techniques for 3D reconstruction.

Technology	Strength	Weakness
MBS	Early adopted	High cost
	Long range and coverage	High minimum distance
	Independent of water turbidity	Low resolution
SBS	Early adopted	High cost
	Long range	Echoes
	Independent of water turbidity	Low resolution
SSS	Good data acquisition rate	High cost
	Independent of water turbidity	Needs constant speed
	Long range	Unknown dimension
IS	Medium to large range	High cost
	Independent of water turbidity	Unknown dimension
LiDAR	Not underwater	Limited to first 15 meters
		Safety constraint
LLS	Medium data acquisition rate	High cost
	Medium range	Safety constraint
	Good performance in scattering waters	
SfM	Simple and inexpensive	Computation demanding
	High accuracy on well-defined targets	Sparse data covering
	Close range	Needs textured scenes
		Unknown scale
SV	Simple and inexpensive	Computation demanding
	High accuracy on well-defined targets	Sparse data covering
	Close range	Low data acquisition rate
PhS	Simple and inexpensive	Limited to smooth surfaces
	Close range	Needs fixed position
VW-SL	High data acquisition rate	Computation demanding
	Close range	Missing data in occlusions and shadows
		Needs fixed position
CW-SL	High data acquisition rate	Computation demanding
	Medium range	Missing data in occlusions and shadows
		Safety constraint if laser source

Table 9 summarizes the main strengths and weaknesses of the solutions surveyed in this article. The comments in the table are quite general, and a number of exceptions may exist. Furthermore, these pros and cons may also be mitigated or increased depending on the application and/or the platform used.

With regard to the use of standard robots as data-gathering platforms, at present, scientists can mount their systems in the payload area, but in general, these systems are independent from the control architecture of the vehicle. As a consequence, the payload and robot work independently; thus, the generation and control of surveys for data sampling missions is still an issue. An adaptive data sampling mission should allow scientists to program the data density in a required area or volume. Then, the controlled robot would only proceed from one mission waypoint to the next only if the data sampling requirement were met. In this way, the resulting data would not lack spatial or temporal resolution. However, work class ROVs or commercial AUVs do not normally have this type of control interface available.

Finally, as was mentioned earlier, to overcome the limitations of each individual sensor type, advanced reconstruction systems can combine various sensors of the same or different natures. This solution can be suited to an underwater robot or to a fleet of them, as using several sensing modalities often requires different speeds and distances from the sea bottom. To make these solutions really functional, much more research effort has to be focused on underwater localization, so that data can be consistently registered and finally integrated in a unique framework.

## Abbreviations

APD: Avalanche photodiode

AUV: Autonomous underwater vehicle

BF: Beam forming

CCD: Charged-couped device

CMOS: Complementary metal-oxide semiconductor

CW-LLS: Continuous wave LLS

DOE: Diffractive optical element

fps; Frames per second

GPS: Global positioning system

GPU: Graphics processor unit

HD: High definition

ICP: Iterative closest point

IMU: Inertial measurement unit

IS: Imaging sonar

KLT: Kanade-Lucas-Tomasi feature tracker

LbSL: Laser-based SL

LED: Light-emitting diode

LiDAR: Light detection and ranging

LLS; Laser line scanning

LS: Laser stripe

MBS: Multibeam sonar

NA: Not available

PG-LLS: Pulse gated LLS

PMT: Photomultiplier tube

PN-MP: Pseudorandom coded modulation pulse

RANSAC: Random sample and consensus

ROV: Remoted operated vehicle

SAM: Smoothing and mapping

SBS: Single beam sonar

SIFT: Scale invariant feature transform

SL: Structured light

SNR: Signal to noise ratio

SONAR: Sound navigation and ranging

SSS: Sidescan sonar

ST-MP: Single tone modulated pulse[SURF] Speeded up robust features

SV: Stereo vision

TOF: Time of flight

UW: Underwater

UWTD: Underwater target detection
